# High Expression of *UBB*, *RAC1*, and *ITGB1* Predicts Worse Prognosis among Nonsmoking Patients with Lung Adenocarcinoma through Bioinformatics Analysis

**DOI:** 10.1155/2020/2071593

**Published:** 2020-10-20

**Authors:** Huan Deng, Yichao Huang, Li Wang, Ming Chen

**Affiliations:** ^1^Department of Radiation Oncology, Cancer Hospital of the University of Chinese Academy of Sciences (Zhejiang Cancer Hospital), Hangzhou 310022, China; ^2^Institute of Cancer Research and Basic Medical (IBMC), Chinese Academy of Sciences, Hangzhou 310022, China; ^3^Zhejiang Key Laboratory of Radiation Oncology, Zhejiang Cancer Hospital, Hangzhou 310022, China; ^4^College of Life Sciences, University of Chinese Academy of Sciences, Beijing 100049, China; ^5^Department of Oncology, Maoming People's Hospital, Maoming 525000, China; ^6^Jiangxi Medical College, Nanchang University, Nanchang 330006, China

## Abstract

**Purpose:**

The molecular mechanism underlying the tumorigenesis and progression of lung adenocarcinoma (LUAD) in nonsmoking patients remains unclear. This study was conducted to select crucial therapeutic and prognostic biomarkers for nonsmoking patients with LUAD.

**Methods:**

Microarray datasets from the Gene Expression Omnibus (GSE32863 and GSE75037) were analyzed for differentially expressed genes (DEGs). Gene Ontology (GO) enrichment analysis of DEGs was performed, and protein-protein interaction network was then constructed using the Search Tool for the Retrieval of Interacting Genes and Cytoscape. Hub genes were then identified by the rank of degree. Overall survival (OS) analyses of hub genes were performed among nonsmoking patients with LUAD in Kaplan-Meier plotter. The Cancer Genome Atlas (TCGA) and The Human Protein Atlas (THPA) databases were applied to verify hub genes. In addition, we performed Gene Set Enrichment Analysis (GSEA) of hub genes.

**Results:**

We identified 1283 DEGs, including 743 downregulated and 540 upregulated genes. GO enrichment analyses showed that DEGs were significantly enriched in collagen-containing extracellular matrix and extracellular matrix organization. Moreover, 19 hub genes were identified, and 12 hub genes were closely associated with OS. Although no obvious difference was detected in *ITGB1*, the downregulation of *UBB* and upregulation of *RAC1* were observed in LUAD tissues of nonsmoking patients. Immunohistochemistry in THPA database confirmed that *UBB* and *ITGB1* were downregulated, while *RAC1* was upregulated in LUAD. GSEA suggested that ribosome, B cell receptor signaling pathway, and cell cycle were associated with *UBB*, *RAC1*, and *ITGB1* expression, respectively.

**Conclusions:**

Our study provides insights into the underlying molecular mechanisms of the carcinogenesis and progression of LUAD in nonsmoking patients and demonstrated *UBB*, *RAC1*, and *ITGB1* as therapeutic and prognostic indicators for nonsmoking LUAD. This is the first study to report the crucial role of *UBB* in nonsmoking LUAD.

## 1. Introduction

Lung cancer (LC) is the most prevalent cancer type, with approximately 228,820 cancer cases and 135,720 death cases in 2020 worldwide, thus causing considerable socioeconomic burdens [1]. Patients with non-small-cell lung cancer (NSCLC) constitute approximately 85% of the total LC cases. Lung adenocarcinoma (LUAD) is the most common histological type of NSCLC [2]. It is widely known that tobacco smoking is a crucial risk factor for LC; however, approximately 20% of LUAD cases occur among nonsmoking patients [3]. Patients with smoking-related LUAD have some altered genes such as *KRAS* and *STK11*. Moreover, these known cancer-related genes may alter simultaneously, leading to a larger tumor mutational burden (TMB) [4, 5]. However, the molecular mechanism underlying the carcinogenesis and progression of nonsmoking-related LUAD remains unclear. In addition, nonsmoking patients are easily ignored as they are not exposed to smoking [6], leading to a high rate of missed diagnosis at early stages. Hence, it is extremely necessary to identify key indicators in the carcinogenesis and development of nonsmoking-related LUAD.

In the past two decades, gene chip technologies and bioinformatics analyses have made great progress in screening genetic alterations at the genome level [7]. These technologies are adopted to find differentially expressed genes (DEGs) that play crucial roles in the occurrence and adverse progression of nonsmoking-related LUAD. However, false-positive rates of independent microarray studies probably weaken the reliability of outcomes [8]. Thus, we selected two microarray datasets on the same platform from the Gene Expression Omnibus (GEO) to acquire DEGs between LUAD tissues of nonsmoking patients and matched adjacent lung tissue samples. Gene Ontology (GO) enrichment analyses of DEGs were then performed, and a protein-protein interaction (PPI) network was constructed for a better understanding of the molecular mechanism underlying tumorigenesis and invasion of nonsmoking-related LUAD. Hub genes were then identified from the PPI network, which are candidates for therapeutic and prognostic biomarkers for nonsmoking-related LUAD. Subsequently, the overall survival (OS) analysis of hub genes was performed. Finally, we validated the findings using The Cancer Genome Atlas (TCGA), The Human Protein Atlas (THPA) database, and Gene Set Enrichment Analysis (GSEA).

## 2. Materials

### 2.1. Microarray Data

GEO (http://www.ncbi.nlm.nih.gov/geo) [9] is a public functional genomics data repository of high-throughput gene expression data, chip, and microarray. All microarray datasets were selected only if they met the following criteria: (1) the topic was on nonsmoking-related LUAD and matched adjacent lung tissues; (2) the platform was GPL6884 Illumina HumanWG-6 v3.0 expression BeadChip; (3) the organism was *Homo sapiens*; (4) the size of matched adjacent lung tissue sample was more than 3; and (5) the last update time was in 2019. In this study, two gene expression profiles (GSE32863 [10] and GSE75037 [11]) from GEO were selected, which met these criteria. GSE32863 included 29 nonsmoking LUAD samples and 30 matched adjacent lung samples, whereas GSE75037 contained 30 nonsmoking LUAD samples and 30 matched adjacent lung tissue samples.

### 2.2. Identification of Differentially Expressed Genes

The DEGs between nonsmoking LUAD and adjacent lung tissues were obtained using GEO2R (http://www.ncbi.nlm.nih.gov/geo/geo2r) [12]. As an interactive web tool, GEO2R allows users to compare at least two datasets in one GEO series for selecting DEGs across experimental conditions. Both adjusted *P* values (adj.*P*) and Benjamini-Hochberg's false discovery rates were adopted to balance the finding of a statistically significant gene and false-positive limitation. Probe sets with no corresponding gene symbols were deleted, whereas genes with at least one probe set were averaged. The cutoff criteria were set as adj.*P* < 0.05 and ∣logFC(fold change) | <1.

### 2.3. Gene Ontology Enrichment Analysis of Differentially Expressed Genes

GO is an important bioinformatics tool for annotating genes and analyzing the biological process of genes [13]. GO enrichment analyses consisted of 3 terms: biological processes (BP), cellular component (CC), and molecular function (MF). All these were performed using the clusterProfiler and GOplot packages in the R software to analyze the functions and signaling pathways of DEGs [14]. *P* value of < 0.05 was regarded statistically significant.

### 2.4. Protein-Protein Interaction Network and Significant Module Construction

The PPI network of DEGs was constructed in the Search Tool for the Retrieval of Interacting Genes (http://string-db.org, version 11.0) [15], and the interaction with a combined score of >0.90 was considered statistically significant. The analysis of the function of PPI provided insights into the mechanism of the development of the disease. Cytoscape (version 3.7.2), a bioinformatics software, was adopted to construct visual networks of molecular interactions [16]. The plug-in Molecular Complex Detection (MCODE) (version 1.4.2) of Cytoscape, a crucial application, was adopted to find closely correlated modules from the PPI network [17]. Genes in significant modules were graphically shown through MCODE plug-in. The selection criteria were set as follows: MCODE score > 5, node score cutoff = 0.2, degree cutoff = 2, *k* − score = 2, and max depth = 100.

### 2.5. Hub Gene Selection and Analysis

The cytoHubba plug-in of Cytoscape was used to calculate the degree rank of hub genes, and hub genes with degrees greater than 20 were selected. The Kyoto Encyclopedia of Genes and Genomes pathway (KEGG) is a crucial database to understand high-level functions and biological systems from large-scale molecular datasets generated by high-throughput experimental technology [18]. To explore their biological function, the BP and KEGG pathway of these hub genes were analyzed and visualized through the ClueGO plug-in [19]. Subsequently, a heat map of hub genes was created and visualized in TCGA database (https://portal.gdc.cancer.gov/). Then, the Kaplan-Meier plotter was used to perform the survival analysis of these hub genes for understanding their prognostic roles (http://kmplot.com/analysis/) [20]. Moreover, TCGA database is an external and relatively authoritative database, which was used for the verification of the difference in the expression levels of hub genes between LUAD and normal lung tissues.

### 2.6. Gene Set Enrichment Analysis

AS a computing approach, GSEA can identify whether previously defined gene sets were statistically significant and concordantly different between the two biological states [21]. Nonsmoking-related LUAD samples were categorized into two groups (high and low expression) by the median expression levels of hub genes. The timing of their expression on many gene sets was then explored to find related KEGG pathways using the molecular signatures database (MSigDB) (c2.cp.kegg.all.v7.1.symbols.gmt) [22]. The number of permutations was set as 1000 times in every analysis. ∣Normalized enrichment score | >1, NOM *P* value < 0.05, and FDR *q* value < 0.25 were considered statistically significant.

## 3. Results

### 3.1. Identification of Differentially Expressed Genes in Nonsmoking Lung Adenocarcinoma

**Figure 1:**
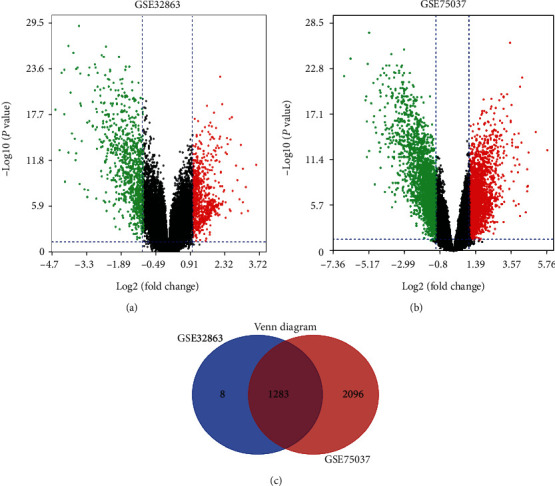
Volcano plot, Venn diagram, and function enrichment analysis of DEGs. The selection process for DEGs with adj.*P* < 0.01 and ∣logFC | <1 in GSE32863 (a) and GSE75037 (b). Upregulated genes are marked in red, and downregulated genes are marked in green. The two datasets display an overlap of 576 DEGs (c). adj.*P*: adjusted *P* values; DEGs: differentially expressed genes; FC: fold change.

The volcano plots illustrated the selection process for DEGs in GSE32863 (Figure 1(a)) and GSE75037 (Figure 1(b)). After normalization of microarray outcomes, we identified DEGs in nonsmoking LUAD and adjacent lung tissues. In addition, the Venn diagram in Figure 1(c) shows that the overlap between both datasets included 1283 DEGs, including 743 downregulated and 540 upregulated genes.

### 3.2. Gene Ontology Enrichment Analysis of Differentially Expressed Genes

**Figure 2:**
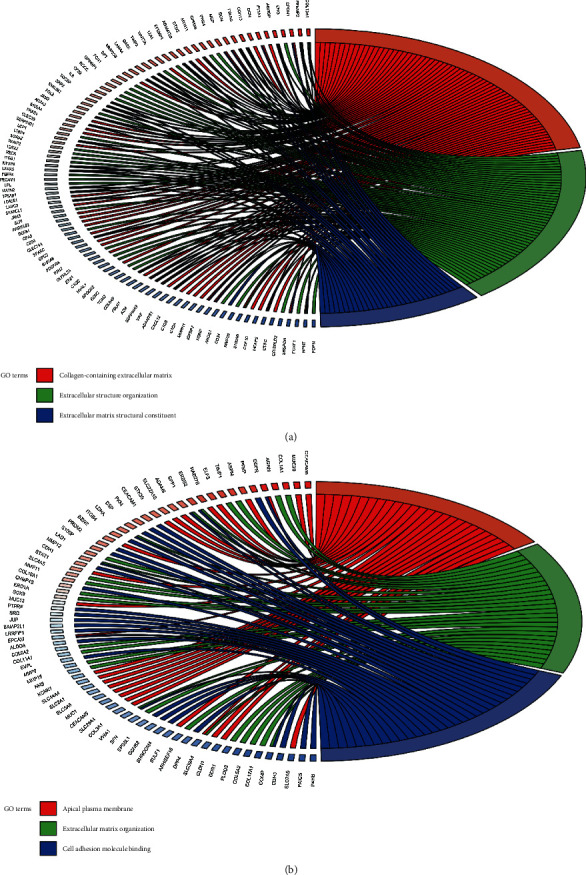
GO enrichment analysis of the DEGs using the clusterProfiler package in the R software. GO enrichment analyses of (a) downregulated DEGs and (b) upregulated DEGs are performed. DEGs: differentially expressed genes; GO: Gene Ontology.

GO enrichment analyses of downregulated DEGs revealed that collagen-containing extracellular matrix (ECM) was considerably enriched in BP, extracellular structure organization in CC, and ECM structural constituent in MF (Figure 2(a)). Moreover, GO enrichment analyses of upregulated DEGs suggested that the ECM organization was primarily enriched in BP, apical plasma membrane in CC, and cell adhesion module binding in MF (Figure 2(b)).

### 3.3. Protein-Protein Interaction Network and Significant Module Construction

**Figure 3:**
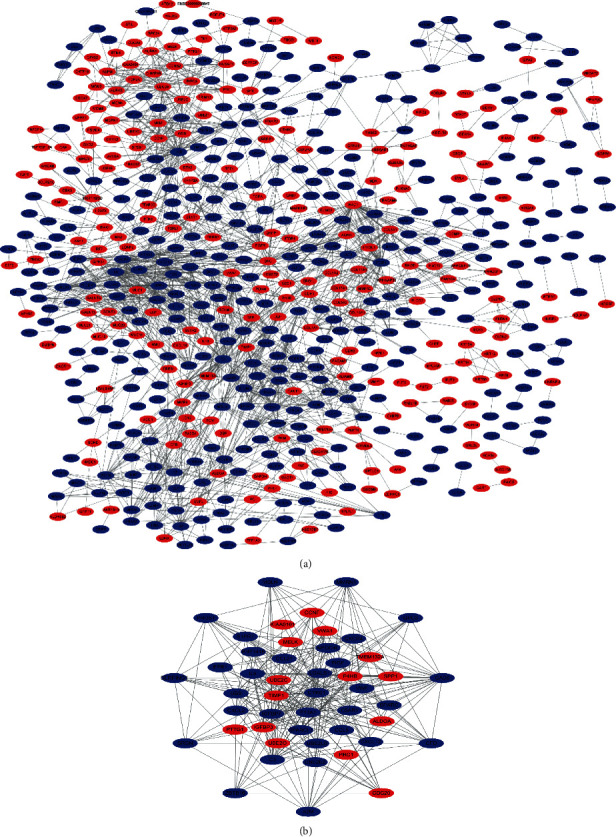
PPI network and the most significant module of DEGs. The PPI network of DEGs is constructed using Cytoscape (a). The most significant module is obtained from the PPI network with 48 nodes and 257 edges (b). Upregulated genes are marked in red and downregulated genes in blue. DEGs: differentially expressed genes; PPI: protein-protein interaction network.

The PPI network of these DEGs is illustrated in Figure 3(a), which consisted of 607 nodes and 1841 edges. The most significant module was then detected through MCODE plug-in. Moreover, this module consisted of 48 nodes and 247 edges, as shown in Figure 3(b), wherein upregulated genes are marked in red and downregulated genes in blue.

### 3.4. Hub Gene Selection and Analysis

**Figure 4:**
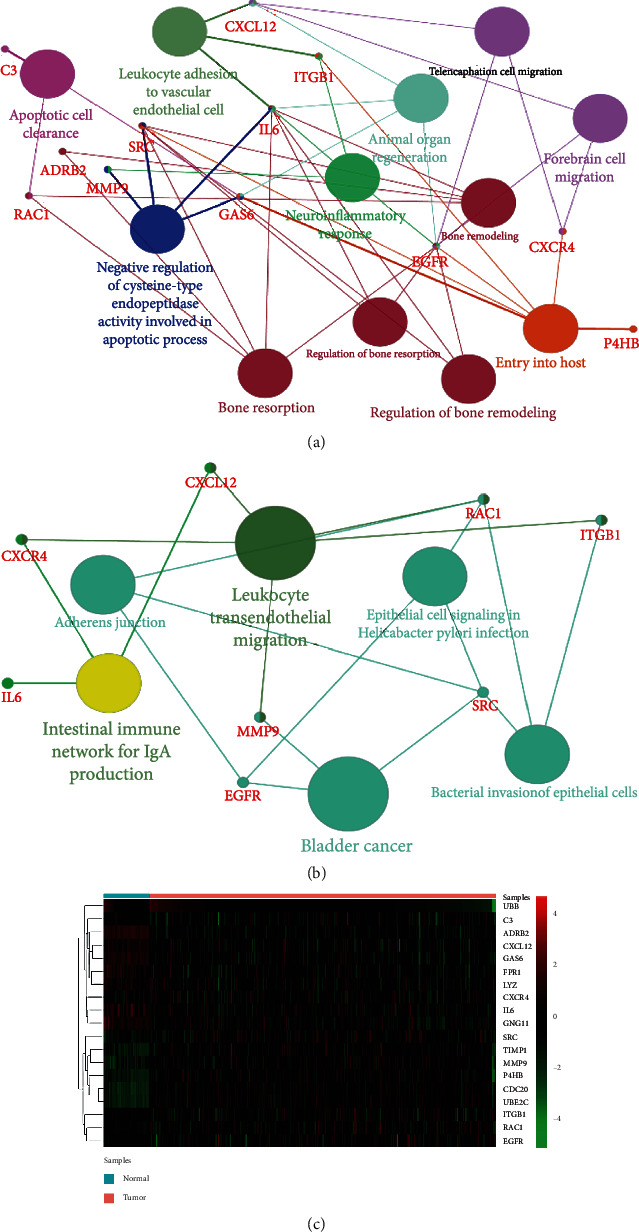
The BP, KEGG pathway analysis, and heat map of hub genes. The BP analysis of hub genes is visualized using the ClueGO plug-in (a). KEGG pathway analysis of hub genes is visualized using the ClueGO plug-in (b). The color depth of nodes refers to the corrected *P* value of ontologies, and the size of nodes refers to the number of genes that participate in the ontologies. *P* < 0.01 is considered statistically significant. Heat map of hub genes is created on the basis of data from TCGA and visualized using pheatmap package (c). Upregulated genes are marked in red and downregulated genes in green. BP: biological process; KEGG: Kyoto Encyclopedia of Genes and Genomes pathway; TCGA: The Cancer Genome Atlas.

**Figure 5:**
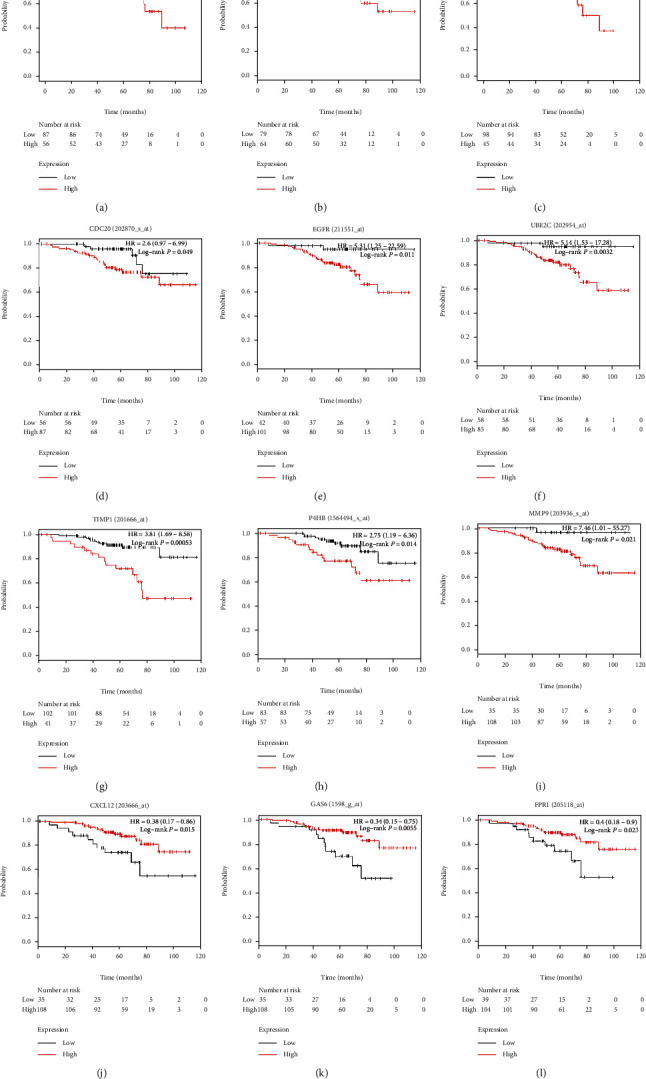
OS analyses of hub genes are performed in a Kaplan-Meier plotter online platform. *UBB*, *RAC1*, *ITGB1*, *CDC20*, *EGFR*, *UBE2C*, *TIMP1*, *P4H8*, and *MMP9* are negatively associated with OS in nonsmoking patients with LUAD (a–h), whereas *CXCL12*, *GAS6*, and *FPR1* are positively correlated with OS (i–k). *P* < 0.05 is considered statistically significant. LUAD: lung adenocarcinoma; OS: overall survival.

A total of 19 DEGs with degrees ≥ 20 were selected as hub genes.  Table 1 shows the gene symbol, degree, full name, and function of these hub genes. BP (Figure 4(a)) and KEGG analysis (Figure 4(b)) of these hub genes are clearly shown in figures. Moreover, a heat map demonstrated the upregulation or downregulation of 19 hub genes in nonsmoking LUAD samples using TCGA dataset (Figure 4(c)). In addition, Figure 5 reveals that *UBB*, *RAC1*, *ITGB1*, *CDC20*, *EGFR*, *UBE2C*, *TIMP1*, *P4H8*, and *MMP9* are negatively correlated with OS in nonsmoking-related LUAD, whereas *CXCL12*, *GAS6*, and *FPR1* are positively correlated with OS. Table S1 displays the hazard ratio (HR), 95% confidence interval (CI), and log-rank *P* value of hub genes.

**Figure 6:**
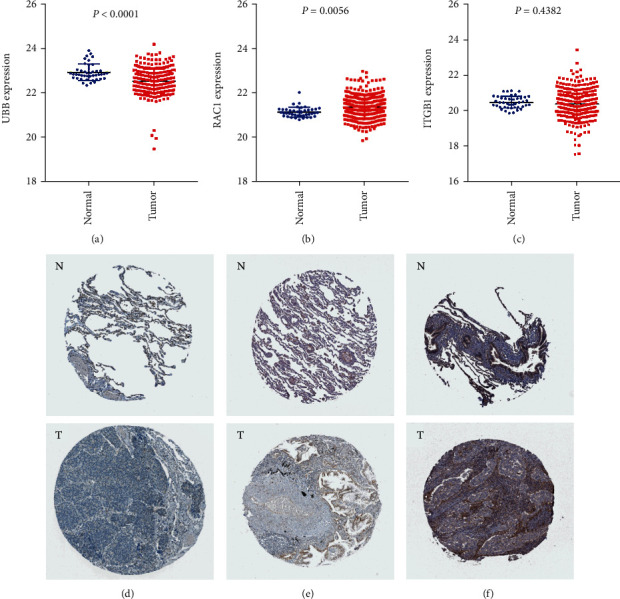
The expression of *UBB*, *RAC1*, and *ITGB1* in normal lung and LUAD tissues using TCGA and THPA databases. The expression of (a) *UBB*, (b) *RAC1*, and (c) *ITGB1* in normal lung and nonsmoking LUAD samples is shown using TCGA database. The results of immunohistochemistry of (d) *UBB*, (e) *RAC1*, and (e) *ITGB1* in normal lung and LUAD tissues using THPA database are displayed. LUAD: lung adenocarcinoma; TCGA: The Cancer Genome Atlas; THPA: The Human Protein Atlas.

*UBB*, *RAC1*, and *ITGB1* had the highest degrees among these hub genes, indicating their pivotal roles in the occurrence and progression of nonsmoking-related LUAD. The overexpression of *UBB*, *RAC1*, and *ITGB1* had worse OS (*P* = 0.00044, *P* = 0.0021, and *P* = 0.00044, respectively), suggesting their potential prognostic implications. Moreover, according to data from TCGA, we found that *UBB* was significantly downregulated (Figure 6(a)), and *RAC1* was upregulated (Figure 6(b)) in nonsmoking-related LUAD. Moreover, *ITGB1* was not significantly different in nonsmoking LUAD and normal lung tissues (Figure 6(c)); however, further studies on this are warranted. Immunohistochemistry (IHC) in THPA database verified that *UBB* (Figure 6(d)) and *ITGB1* (Figure 6(e)) were downregulated, and *RAC1* (Figure 6(f)) was upregulated in LUAD.

### 3.5. Gene Set Enrichment Analysis

**Figure 7:**
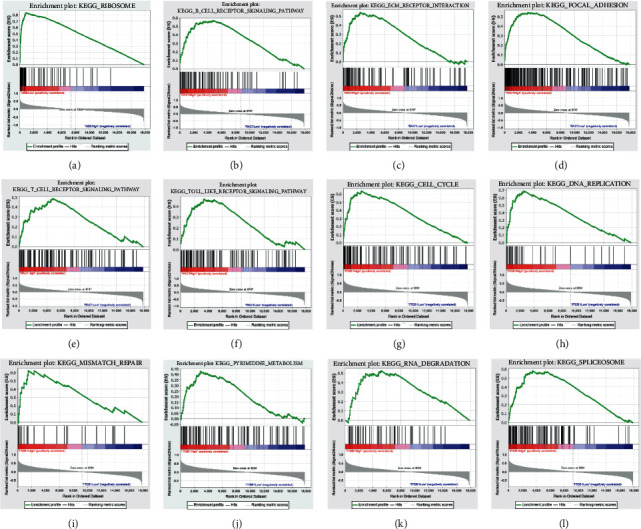
Enrichment plots by GSEA. Relative pathways associated with the expression of (a) *UBB*, (b–f) *RACI*, and (g–l) *ITGB1* are displayed. GSEA: Gene Set Enrichment Analysis; KEGG: Kyoto Encyclopedia of Genes and Genomes pathway.

GSEA showed that ribosome was associated with UBB expression (Figure 7(a)). Furthermore, it suggested that ECM receptor interaction, B cell receptor signaling pathway, T cell receptor signaling pathway, toll-like receptor signaling pathway, and focal adhesion were correlated with *RAC1* expression (Figures 7(b)–7(f)). In addition, cell cycle, spliceosome, DNA replication, RNA degradation, mismatch repair, and pyrimidine metabolism were associated with *ITGB1* expression (Figures 7(g)–7(m)). The detailed outcomes of the analysis were shown in Table 2.

## 4. Discussion

LC commonly has a high mortality rate and results in great socioeconomic pressure for patients, families, and countries. Certainly, smoking contributes to the occurrence and development of LC. However, one in five LUAD cases occurs among patients who do not smoke [3]. The alterations of some genes, including *EGFR*, *ERBB2*, *ALK*, *ROS1*, and *RET*, are evidently associated with the occurrence and progression of LUAD [23]. However, the underlying molecular mechanism of nonsmoking-related LUAD remains unclear. Therefore, it is essential to identify crucial biomarkers for understanding the molecular mechanism of nonsmoking-related LUAD. Microarray technology is available for finding new biomarkers, which will be the basis of future studies on the potential mechanism of nonsmoking-related LUAD.

We analyzed two microarray datasets to obtain DEGs between nonsmoking LUAD and matched adjacent lung tissues. A total of 1283 DEGs were selected, containing 743 downregulated and 540 upregulated genes. Function enrichment analyses manifested that DEGs were mainly enriched in collagen-containing ECM, ECM organization, and apical plasma membrane. Moreover, 19 hub genes with degrees greater than 20 were selected from the PPI network: *UBB*, *RAC1*, *ITGB1*, *SRC*, *C3*, *IL6*, *CDC20*, *EGFR*, *UBE2C*, *TIMP1*, *GNG11*, *CXCL12*, *GAS6*, *P4HB*, *CXCR4*, *FPR1*, *ADRB2*, *LYZ*, and *MMP9*. BP enrichment analysis of these hub genes suggested that apoptotic cell clearance, negative regulation of cysteine-type endopeptidase activity involved in apoptotic process, and leukocyte adhesion to vascular endothelial cell were primarily enriched, and the most enriched KEGG pathway was leukocyte transendothelial migration, bladder cancer, and intestinal immune network for IgA production. Among 19 hub genes, 12 hub genes were closely associated with poorer OS in nonsmoking patients with LUAD. From the results of GO enrichment analysis, KEGG pathway analysis, and survival analysis and degree rank, *UBB*, *RAC1*, and *ITGB1* were believed to be the core genes in the occurrence and development of nonsmoking-related LAUD at the molecular level. Besides, IHC outcomes in THPA database confirmed that *UBB* and *ITGB1* were downregulated, and *RAC1* was upregulated in nonsmoking-related LUAD.

This study was the first one to report the key role of *UBB* in nonsmoking-related LUAD. Ubiquitin B (*UBB*) is a crucial member of gene families encoding ubiquitin. Ubiquitin is involved in several cellular processes, and aberrant events in ubiquitin-mediated processes promote the carcinogenesis and progression of NSCLC [24]. *UBB* is strongly suppressed in some cancers, including endometrial carcinoma and ovarian cancer [25]. Tang et al. revealed that ubiquitin was highly expressed in NSCLC tissues; however, increased ubiquitin was attributed to the increased transcripts of ubiquitin C (UBC) rather than *UBB*. Moreover, they showed that no significant difference was observed in the *UBB* mRNA level between NSCLC and normal lung tissues (*P* = 0.167) [26]. This finding contradicted our results, and there can be two probable reasons for this difference. First, Tang et al. compared NSCLC and normal lung tissues, whereas we compared nonsmoking-related LUAD and normal lung tissues, thus suggesting higher accuracy of our study. Second, this study compared nonsmoking-related LUAD and matched adjacent lung tissues with similar features instead of unmatched normal lung tissues, revealing higher reliability of our study. However, future studies on the expression level of *UBB* are warranted to confirm findings. Additionally, according to the survival analysis, *UBB* may play a crucial prognostic role in nonsmoking-related LUAD.

As one member of the RAS superfamily of small GTP-binding proteins, Rac Family Small GTPase 1, *RAC1*, can interact with effector proteins, and downstream kinases were activated to regulate multiple cellular processes [27]. In addition, *RAC1* improved nuclear factor kappa B activity to regulate cell proliferation and migration in NSCLC [28]. *RAC1* is upregulated in various cancers, such as LUAD, breast cancer, and kidney cancer [29], and the overexpression of *RAC1* is frequently reported to be associated with worse prognosis [30]. One study suggested that high expression of *RAC1* plays a key role in the epithelial-mesenchymal transition and malignant progression in LUAD [31]. In addition, KIF18B promotes cell proliferation and invasion through activating *RAC1* and mediating the AKT/mTOR signaling pathway in LUAD, indicating the crucial role of *RAC1* in the cell proliferation and adverse progression of LUAD [32]. Similarly, Li et al. showed that intracellular mature interleukin 37 can inhibit tumor metastasis through inhibiting *RAC1* activation [33], suggesting a crucial role of *RAC1* in the tumor metastasis. In addition, *RAC1* may predict the prognosis of nonsmoking patients with LUAD in light of our survival analysis.

Integrin subunit beta 1 (*ITGB1*) encodes the beta subunit of integrins, which is a heterodimeric cell surface receptor and participates in the carcinogenesis, migration, and invasion of LUAD [34]. *ITGB1* is abnormally expressed in several cancers, including LUAD and breast cancer [35]. MicroRNA- (miR-) 134 inhibits the migration and metastasis by targeting *ITGB1* in NSCLC [36]. Similarly, high expression of miR-493–5p is correlated with better survival of NSCLC via targeting *ITGB1* [37], indicating the crucial role of *ITGB1* in the carcinogenesis and worse prognosis of NSCLC. Zheng et al. observed that *ITGB1* is a predictive biomarker of NSCLC after matching clinical factors (odds ratio (OR) = 1.31, 95% CI: 1.10–1.55) [38]. Our survival analysis also confirmed the prognostic value of *ITGB1*. Furthermore, GSEA revealed that ribosome was correlated with *UBB* expression; ECM receptor interaction, B cell receptor signaling pathway, and T cell receptor signaling pathway with *RAC1* expression; and cell cycle, spliceosome, and DNA replication with *ITGB1* expression. From these findings, we concluded that *UBB*, *RAC1*, and *ITGB1* were therapeutic and prognostic biomarkers of nonsmoking-related LUAD.

Undeniably, this study was not the first one to select pivotal genes and pathways in nonsmoking-related LUAD using bioinformatics methods. In fact, three similar analyses were previously published [39–41]. However, previous studies had several differences and/or disadvantages compared with this study. First, two analyses [40, 41] compared NSCLC/LC in nonsmoking females and normal lung tissues rather than nonsmoking LUAD, suggesting lower accuracy of the study. Second, LUAD/NSCLC/LC in nonsmoking females and normal lung tissues were compared in the previous studies [39–41], whereas male patients with LUAD were excluded. However, this study has several advantages compared with previous studies: (1) this study included both male and female patients with nonsmoking LUAD, which may have potential benefits for nonsmoking male patients with LUAD; (2) this study compared nonsmoking-related LUAD and matched adjacent lung tissues with similar features, thus having higher reliability; (3) this study revealed some novel findings regarding *UBB*, *UBE2C*, *GAS6*, and *P4HB*; and (4) some advanced analyses such as GSEA were also performed in this study.

This study also has some limitations. (1) Only two datasets were included. Several similar microarray datasets exist, but they did not meet the selection criteria of our study. Therefore, to decrease the bias, these similar datasets had to be excluded from this study. (2) The included datasets did not reveal detailed information about survival time; therefore, survival analysis of hub genes had to be performed using the Kaplan-Meier plotter. (3) These findings were not verified by performing experiments, which was warranted in future studies.

## 5. Conclusion

Our study was conducted to select DEGs that may correlate with the carcinogenesis and malignant invasion of nonsmoking-related LUAD. A total of 19 hub genes were selected from the PPI network, and 12 hub genes correlated with the prognosis of nonsmoking-related LUAD. Furthermore, *UBB*, *RAC1*, and *ITGB1* were potential therapeutic and prognostic indicators of nonsmoking-related LUAD. Moreover, this study is the first to report the key role of *UBB* in nonsmoking-related LUAD. This study provided evidence for future genomic-based individualized treatments of LUAD in nonsmoking patients. However, future studies are warranted to explore further the biological relationships among these DEGs in nonsmoking-related LUAD.

## Figures and Tables

**Table 1:** Functional roles of 19 hub genes with degrees > 20.

No.	Gene symbol	Degree	Full name	Function
1	UBB	51	Ubiquitin B	UBB is lowly expressed in some cancers, such as nonsmoking LUAD and endometrial carcinoma.
2	RAC1	50	Rac Family Small GTPase 1	RAC1 serves a key role in the EMT, cell proliferation, and worse invasion in LUAD.
3	ITGB1	42	Integrin subunit beta 1	ITGB1 encodes the beta subunit of integrins and participates in the carcinogenesis and progression of LUAD.
4	SRC	40	SRC protooncogene, nonreceptor tyrosine kinase	SRC participates in the cell proliferation, migration, and invasion of LUAD.
5	C3	40	Complement C3	C3 is obviously altered in serum among patients with LUAD and C3 may be a candidate diagnostic biomarker.
6	IL6	33	Interleukin 6	IL6 promotes Kras driven of the carcinogenesis of LUAD and induces the resistance of gefitinib in EGFR-mutant LC.
7	CDC20	33	Cell division cycle 20	CDC20 is associated with protein ubiquitination and modification and promotes the progression of NSCLC.
8	EGFR	32	Epidermal growth factor receptor	EGFR mutation is the driven alteration of nonsmoking LUAD and participates in the cell proliferation in LUAD.
9	UBE2C	29	Ubiquitin-conjugating enzyme E2 C	UBE2C contributes to the EMT, cell proliferation, and malignant invasion of LUAD.
10	TIMP1	28	TIMP metallopeptidase inhibitor 1	TIMP1 participates in the progression and inhibits the apoptosis of tumor cells in LUAD.
11	GNG11	26	G protein subunit gamma 11	Low expression of GNG11 is correlated with poorer prognosis among woman patients with nonsmoking LC.
12	CXCL12	26	C-X-C motif chemokine ligand 12	CXCL12/CXCR4 plays an important role in the propagation of NSCLC.
13	GAS6	25	Growth arrest specific 6	AXL/GAS6 axis contributes to cell migration in NSCLC, which is a candidate prognostic biomarker in NSCLC.
14	P4HB	25	Prolyl 4-hydroxylase subunit beta	P4HB participates in the invasion and metastasis of gastric cancer.
15	CXCR4	24	C-X-C motif chemokine receptor 4	CXCR4 serves a procarcinogenic role by interacting with CXCL12 in NSCLC.
16	FPR1	22	Formyl peptide receptor 1	FPR1 induces the translocation of NF-*κ*B to promote the progression by upregulating IL6 and IL8 in cervical cancer.
17	ADRB2	22	Adrenoceptor beta 2	ADRB2 is overexpressed in LUAD, which promotes the adverse progression in LUAD.
18	LYZ	21	Lysozyme	Hypermethylated LYZ is observed in gastric cancer.
19	MMP9	21	Matrix metallopeptidase 9	MMP9 regulates the cell proliferation and metastasis of LUAD.

Abbreviations: LC: lung cancer; NSCLC: non-small-cell lung cancer; LUAD: lung adenocarcinoma; TSA: trichostatin A; EMT: epithelial-mesenchymal transition; NF-*κ*B: nuclear factor kappa B.

**Table 2:** KEGG pathways associated with the expression of UBB, RAC1, and ITGB1 using GSEA.

Gene	Name	ES	NES	NOM *P* value	FDR *q* value
UBB	KEGG_ribosome	0.84	2.00	≤0.001	0.012

RAC1	KEGG_ECM_receptor_interaction	0.54	1.74	0.032	0.100
	KEGG_B_cell_receptor_signaling_pathway	0.57	1.79	0.006	0.111
	KEGG_T_cell_receptor_signaling_pathway	0.49	1.64	0.026	0.132
	KEGG_toll_like_receptor_signaling_pathway	0.47	1.64	0.019	0.133
	KEGG_focal_adhesion	0.54	1.81	0.010	0.188

ITGB1	KEGG_cell_cycle	0.64	2.09	≤0.001	0.005
	KEGG_spliceosome	0.59	1.97	≤0.001	0.022
	KEGG_DNA_replication	0.59	1.82	0.018	0.056
	KEGG_RNA_degradation	0.53	1.83	≤0.001	0.066
	KEGG_mismatch_repair	0.62	1.68	0.032	0.204
	KEGG_pyrimidine_metabolism	0.43	1.65	0.010	0.232

Abbreviations: GSEA: Gene Set Enrichment Analysis; NES: normalized enrichment score; NOM: nominal; FDR: false discovery rate.

## Data Availability

The datasets downloaded and analyzed during the current study are available on the GEO database: GSE32863, https://www.ncbi.nlm.nih.gov/geo/query/acc.cgi?acc=GSE32863;GSE75037, https://www.ncbi.nlm.nih.gov/geo/query/acc.cgi?acc=GSE75037.

## Abbreviations

LC: Lung cancer
NSCLC: Non-small-cell lung cancer
LUAD: Lung adenocarcinoma
TMB: Tumor mutation burden
DEGs: Differentially expressed genes
GEO: Gene Expression Omnibus
TCGA: The Cancer Genome Atlas
THPA: The Human Protein Atlas
GO: Gene Ontology
KEGG: Kyoto Encyclopedia of Genes and Genomes pathway
PPI: Protein-protein interaction network
adj.*P*: Adjusted *P* values
FC: Fold change
STRING: Search Tool for the Retrieval of Interacting Genes
MCODE: Molecular Complex Detection
KM: Kaplan-Meier plotter
BP: Biological processes
CC: Cellular component
MF: Molecular function
OS: Overall survival
HR: Hazard ratio
CI: Confidence interval
GSEA: Gene Set Enrichment Analysis
miRNA: MicroRNA
OR: Odds ratio.

## Supplementary Materials

Supplementary Materials Table S1: overall survival analysis of hub genes using K-M plotter.

## References

[1] Siegel R. L., Miller K. D., Jemal A. (2020). Cancer statistics, 2020. *CA: A Cancer Journal for Clinicians*.

[2] Chen Z., Fillmore C. M., Hammerman P. S., Kim C. F., Wong K. K. (2014). Non-small-cell lung cancers: a heterogeneous set of diseases. *Nature Reviews Cancer*.

[3] Chiu M., Lipka M. B., Bhateja P., Fu P., Dowlati A. (2020). A detailed smoking history and determination of MYC status predict response to checkpoint inhibitors in advanced non-small cell lung cancer. *Translational Lung Cancer Research*.

[4] Zhang H., Deng Y. M., Chen Z. C. (2020). Clinical significance of tumor mutation burden and DNA damage repair in advanced stage non-small cell lung cancer patients. *European Review for Medical and Pharmacological Sciences*.

[5] Cancer Genome Atlas Research Network (2014). Comprehensive molecular profiling of lung adenocarcinoma. *Nature*.

[6] Pros E., Saigi M., Alameda D. (2020). Genome-wide profiling of non-smoking-related lung cancer cells reveals common RB1 rearrangements associated with histopathologic transformation in EGFR-mutant tumors. *Annals of Oncology*.

[7] Dawany N. B., Dampier W. N., Tozeren A. (2011). Large-scale integration of microarray data reveals genes and pathways common to multiple cancer types. *International Journal of Cancer*.

[8] Manoli T., Gretz N., Gröne H. J., Kenzelmann M., Eils R., Brors B. (2006). Group testing for pathway analysis improves comparability of different microarray datasets. *Bioinformatics*.

[9] Barrett T., Wilhite S. E., Ledoux P. (2013). NCBI GEO: archive for functional genomics data sets--update. *Nucleic Acids Research*.

[10] Selamat S. A., Chung B. S., Girard L. (2012). Genome-scale analysis of DNA methylation in lung adenocarcinoma and integration with mRNA expression. *Genome Research*.

[11] Girard L., Rodriguez-Canales J., Behrens C. (2016). An expression signature as an aid to the histologic classification of non-small cell lung cancer. *Clinical Cancer Research*.

[12] Davis S., Meltzer P. S. (2007). GEOquery: a bridge between the Gene Expression Omnibus (GEO) and BioConductor. *Bioinformatics*.

[13] Ashburner M., Ball C. A., Blake J. A. (2000). Gene ontology: tool for the unification of biology. The Gene Ontology Consortium. *Nature Genetics*.

[14] Yu G., Wang L. G., Han Y., He Q. Y. (2012). clusterProfiler: an R package for comparing biological themes among gene clusters. *OMICS*.

[15] Franceschini A., Szklarczyk D., Frankild S. (2013). STRING v9.1: protein-protein interaction networks, with increased coverage and integration. *Nucleic Acids Research*.

[16] Reimand J., Isserlin R., Voisin V. (2019). Pathway enrichment analysis and visualization of omics data using g:Profiler, GSEA, Cytoscape and EnrichmentMap. *Nature Protocols*.

[17] Bandettini W. P., Kellman P., Mancini C. (2012). MultiContrast Delayed Enhancement (MCODE) improves detection of subendocardial myocardial infarction by late gadolinium enhancement cardiovascular magnetic resonance: a clinical validation study. *Journal of Cardiovascular Magnetic Resonance*.

[18] Kanehisa M., Goto S. (2000). KEGG: kyoto encyclopedia of genes and genomes. *Nucleic Acids Research*.

[19] Mlecnik B., Galon J., Bindea G. (2019). Automated exploration of gene ontology term and pathway networks with ClueGO-REST. *Bioinformatics*.

[20] Győrffy B., Surowiak P., Budczies J., Lánczky A. (2013). Online survival analysis software to assess the prognostic value of biomarkers using transcriptomic data in non-small-cell lung cancer. *PLoS One*.

[21] Subramanian A., Tamayo P., Mootha V. K. (2005). Gene set enrichment analysis: a knowledge-based approach for interpreting genome-wide expression profiles. *Proceedings of the National Academy of Sciences of the United States of America*.

[22] Liberzon A., Subramanian A., Pinchback R., Thorvaldsdottir H., Tamayo P., Mesirov J. P. (2011). Molecular signatures database (MSigDB) 3.0. *Bioinformatics*.

[23] Dagogo-Jack I., Robinson H., Mino-Kenudson M. (2019). Expediting comprehensive molecular analysis to optimize initial treatment of lung Cancer patients with minimal smoking history. *Journal of Thoracic Oncology*.

[24] Zhang K., Yang L., Wang J. (2019). Ubiquitin-specific protease 22 is critical to in vivo angiogenesis, growth and metastasis of non-small cell lung cancer. *Cell Communication and Signaling*.

[25] Haakonsen D. L., Rape M. (2017). Ubiquitin levels: the next target against gynecological cancers?. *The Journal of Clinical Investigation*.

[26] Tang Y., Geng Y., Luo J. (2015). Downregulation of ubiquitin inhibits the proliferation and radioresistance of non-small cell lung cancer cells in vitro and in vivo. *Scientific Reports*.

[27] Guan X., Guan X., Dong C., Jiao Z. (2020). Rho GTPases and related signaling complexes in cell migration and invasion. *Experimental Cell Research*.

[28] Gastonguay A., Berg T., Hauser A. D., Schuld N., Lorimer E., Williams C. L. (2012). The role of Rac1 in the regulation of NF-*κ*B activity, cell proliferation, and cell migration in non-small cell lung carcinoma. *Cancer Biology & Therapy*.

[29] De P., Aske J. C., Dey N. (2019). RAC1 takes the lead in solid tumors. *Cell*.

[30] Bid H. K., Roberts R. D., Manchanda P. K., Houghton P. J. (2013). RAC1: an emerging therapeutic option for targeting cancer angiogenesis and metastasis. *Molecular Cancer Therapeutics*.

[31] Seiz J. R., Klinke J., Scharlibbe L. (2020). Different signaling and functionality of Rac1 and Rac1b in the progression of lung adenocarcinoma. *Biological Chemistry*.

[32] Ji Z., Pan X., Shang Y., Ni D. T., Wu F. L. (2019). KIF18B as a regulator in microtubule movement accelerates tumor progression and triggers poor outcome in lung adenocarcinoma. *Tissue & Cell*.

[33] Li Y., Zhao M., Guo C. (2018). Intracellular mature IL-37 suppresses tumor metastasis via inhibiting Rac1 activation. *Oncogene*.

[34] Xia Z. J., Hu W., Wang Y. B., Zhou K., Sun G. J. (2014). Expression heterogeneity research of ITGB3 and BCL-2 in lung adenocarcinoma tissue and adenocarcinoma cell line. *Asian Pacific Journal of Tropical Medicine*.

[35] Sun Q., Zhou C., Ma R. (2018). Prognostic value of increased integrin-beta 1 expression in solid cancers: a meta-analysis. *Oncotargets and Therapy*.

[36] Qin Q., Wei F., Zhang J., Li B. (2017). miR-134 suppresses the migration and invasion of non-small cell lung cancer by targeting ITGB1. *Oncology Reports*.

[37] Liang Z., Kong R., He Z. (2017). High expression of miR-493–5p positively correlates with clinical prognosis of non small cell lung cancer by targeting oncogene ITGB1. *Oncotarget*.

[38] Zheng W., Jiang C., Li R. (2016). Integrin and gene network analysis reveals that ITGA5 and ITGB1 are prognostic in non-small-cell lung cancer. *Oncotargets and Therapy*.

[39] Wang H., Zhang Z., Xu K., Wei S., Li L., Wang L. (2019). Exploration of estrogen receptor-associated hub genes and potential molecular mechanisms in non-smoking females with lung adenocarcinoma using integrated bioinformatics analysis. *Oncology Letters*.

[40] Shi K., Li N., Yang M., Li W. (2019). Identification of key genes and pathways in female lung cancer patients who never smoked by a bioinformatics analysis. *Journal of Cancer*.

[41] Zhou W., Yin M., Cui H. (2015). Identification of potential therapeutic target genes and mechanisms in non-small-cell lung carcinoma in non-smoking women based on bioinformatics analysis. *European Review for Medical and Pharmacological Sciences*.

