# Supplementary material for: High Expression of UBB, RAC1, and ITGB1 Predicts Worse Prognosis among Nonsmoking Patients with Lung Adenocarcinoma through Bioinformatics Analysis
Source: Biomed Res Int. 2020 Oct 20;2020:2071593. doi: 10.1155/2020/2071593 (PMC7593752; doi:10.1155/2020/2071593)
Supplement: Supplementary Materials — Table S1: overall survival analysis of hub genes using K-M plotter. [file 2071593.f1.docx]

Table S1. Overall survival analysis of hub genes using K-M plotter.

| **Gene Symbol** | **HR** | **95%CI** | **Log-rank P-Value** |
| --- | --- | --- | --- |
| UBB | 1.03 | 0.46-2.29 | 0.950 |
| RAC1 | 3.87 | 1.53-9.76 | 0.0021 |
| ITGB1 | 3.95 | 1.73-9.04 | 0.00044 |
| SRC | 1.99 | 0.88-4.48 | 0.090 |
| C3 | 0.62 | 0.28-1.39 | 0.240 |
| IL6 | 1.61 | 0.68-3.78 | 0.270 |
| CDC20 | 2.60 | 0.97-6.99 | 0.049 |
| EGFR | 5.31 | 1.25-22.59 | 0.011 |
| UBE2C | 5.14 | 1.53-17.28 | 0.0032 |
| TIMP1 | 3.81 | 1.69-8.58 | 0.00053 |
| GNG11 | 0.59 | 0.26-1.31 | 0.190 |
| CXCL12 | 0.38 | 0.17-0.86 | 0.015 |
| GAS6 | 0.34 | 0.15-0.75 | 0.0055 |
| P4HB | 2.75 | 1.19-6.36 | 0.014 |
| CXCR4 | 0.50 | 0.22-1.22 | 0.084 |
| FPR1 | 0.40 | 0.18-0.90 | 0.023 |
| ADRB2 | 0.46 | 0.21-1.05 | 0.059 |
| LYZ | 0.67 | 0.30-1.50 | 0.330 |
| MMP9 | 7.46 | 1.01-55.27 | 0.021 |

**Abbreviations:** K-M plotter: Kaplan‑Meier plotter; HR: hazard ratio; CI: confidence interval.
